# Medical Opinion and Movement

**Published:** 1907-10-19

**Authors:** 


### Medical Opinion and Movement.

The Registrar-General's returns show that during the quarter ending on September 28 the death-rate in the 76 large English towns was exceptionally low. It amounted to 12.3 per 1,000 per annum; that is, 3.7 below the mean rate in these towns for the corresponding quarter of the four years 1903-1906. A striking and satisfactory feature in this reduced mortality is the decline in infant mortality. During the summer quarter of last year deaths of infants under one year of age amounted to 23,065 in the 76 towns, or 209 per annum per 1,000 births. For the summer quarter of this year the figures are 11,611, or 110 per 1,000 births. A low mean temperature undoubtedly contributed largely to this fall in mortality, especially to that from diarrhœa. Deaths from diarrhœa in the 76 towns during last summer were 14,312 and from the same cause this summer only 2,339. With due allowance for the favourable meteorological conditions, there seems ground for belief that the decrease in infant mortality may also be in some measure due to the efforts which have been made to diminish the waste of infant life. In Huddersfield, where special preventive measures have been adopted, the rate of infant mortality has fallen from 220 per 1,000 last year to 62 per 1,000 in 1907.

Hitherto it has been generally recognised that removal of the parotid gland involved serious injury to the facial nerve, followed by facial paralysis of that side of the face. Surgeons always warn their patients of the probable consequences of operation in cases where removal is a necessity, and if possible avoid the operation altogether. It is therefore of considerable interest to record that two cases of parotid tumour have recently been treated surgically, and by careful dissection of the branches of the nerve it has been preserved almost intact, with the result that only temporary paralysis has followed the operation. The first case is recorded by Mr. J. Carwardine, of the Bristol Royal Infirmary. A girl aged 18 had a mixed tumour of the parotid which was successfully shelled out by Mr. Carwardine. The tumour, however, returned two months later, necessitating the total removal of the gland. By careful dissection the various branches of the facial nerve were freed from the gland first, and then the whole gland was excised. Facial paralysis followed, but signs of recovery began two months later, and within a year the patient had good control of the facial muscles of that side. The second case is recorded by Mr. Leveson Gower Gunn, of Dublin. In this case also complete excision of the parotid was performed after dissection of the nerve. Facial paralysis followed, but within five weeks it had so far disappeared that no difference could be detected between the two sides of the face.

The triumphs of modern medicine in the domains of tropical disease are widely known to the public as well as to the profession. It may be doubted, however, whether it is as widely appreciated with what toil and industry the foundations of these triumphs have been laid. The secrets of success have been penetrated by laborious scientific investigation, not haphazard or by chance. In a brief monograph by Dr. C. F. Fothergill, of the London School of Tropical Medicine, it is possible to read much between the lines as to the methods that have led to the conclusions which he enunciates. The essay deals solely with the value and significance of blood examinations in tropical medicine, given in explicit accounts of the blood characters of each disease: technique is outside the author's scope, and diagnosis alone is kept in view. The variations found in the blood picture of each disease are fully dealt with, and both in this respect and in the careful schemes by which distinct conditions may be differentiated, it is evident on what a mass of data the assertions of hœmologists are rested. A collection of thirty illustrative cases is reported with admirable restraint, but in just sufficient detail to point the moral of each. As a lucid and well-arranged statement of our knowledge at the present time, Dr. Fothergill's booklet fulfils his aims in a thoroughly adequate manner.

Now that the medical inspection of school children has become law, it is of the utmost importance that the medical profession should direct their attention to all questions relating to school hygiene, and should use their influence to render the application of the law as practicable and efficacious as possible. Not the least important of these questions is the influence of school life upon the incidence of tuberculosis. Dr. John Lowman, of Cleveland, has recently expressed his views on the subject. His programme is very comprehensive, and the measures which he advocates are so elaborate that one can hardly hope to see the adoption of even a tithe of them for a long time to come. Nevertheless his scheme is sufficiently practical to serve as an ideal to pursue, in devising means of prevention of the spread of the disease among school children. In the first place he considers that infected children and those living in infected houses should be separated out, and that the contagious cases should be placed in special sanatoriums, and the others in separate schools. These children should also be separated from their healthy comrades during the vacations by referring them to the special consideration of "outing societies." The utmost precautions should be used for the protection of the teachers. Furthermore, he advocates the introduction of systematic courses on hygiene and tuberculosis into the curriculum of the schools. This last provision is already being urged upon the authorities, and we hope ere long to see it incorporated into the general scheme of hygiene for the elementary schools of this country.

